# Effect of Mineral Composition and Particle Size on the Failure Characteristics and Mechanisms of Marble in the China Jinping Underground Laboratory

**DOI:** 10.3390/ma17102290

**Published:** 2024-05-12

**Authors:** Hong Xu, Peiqi Jing, Guangliang Feng, Zhen Zhang, Quan Jiang, Jie Yan

**Affiliations:** 1 National Engineering Research Center of Highway Maintenance Technology, Changsha University of Science & Technology, Changsha 410114, China; 2 School of Civil Engineering, Changsha University of Science & Technology, Changsha 410114, China; 3 State Key Laboratory of Geomechanics and Geotechnical Engineering, Institute of Rock and Soil Mechanics, Chinese Academy of Sciences, Wuhan 430071, China

**Keywords:** grain size, mineral composition, true triaxial test, mechanical properties, failure mechanism

## Abstract

In deep underground engineering, the deformation, failure characteristics, and mechanism of surrounding rock under the influence of grain sizes and mineral compositions are not clear. Based on CJPL-II variously colored marbles, the differences in grain size and mineral composition of the marble were analyzed by thin-section analysis and XRD tests, and the effect of intermediate principal stress on the mechanical properties of marble was investigated. Both SEM and microfracture analysis were coupled to reveal the failure mechanisms. The results highlight that the crack initiation strength, damage strength, peak strength, and elasticity modulus of Jinping marble exhibit an increasing trend with an increase in intermediate principal stress, while the peak strain initially increases and subsequently decreases. Moreover, this study established negative correlations between marble strength, brittleness characteristics, and fracture angle with grain size, whereas positive correlations were identified with the content of quartz, sodium feldspar, and the magnitude of the intermediate principal stress. The microcrack density in marble was found to increase with larger grain sizes and decrease with elevated quartz and sodium feldspar content, as well as with increasing intermediate principal stress. Notably, as the intermediate principal stress intensifies and grain size diminishes, the transgranular tensile failure of marble becomes more conspicuous. These research findings contribute to the effective implementation of disaster prevention and control strategies.

## 1. Introduction

With the development of the social economy, engineering construction is being carried out continuously into the deep part of the earth. Deep engineering rock masses are mostly in true triaxial high-geostress environments [1,2,3,4,5,6], which are likely to cause engineering disasters, such as rock bursts [7,8,9,10,11], spalling [12,13], and large deformation of the surrounding rock [14], during excavation. Rock is an anisotropic aggregate formed by the cementation and contact of mineral particles [15], and its macroscopic mechanical properties are significantly affected by the grain size and mineral composition. The engineering disasters triggered by the excavation unloading of rock mass with different grain sizes and mineral compositions under true triaxial conditions are different. Consequently, studying the mechanical properties and failure mechanisms of rocks with different grain sizes and mineral compositions under true triaxial conditions is of great significance.

Regarding the study of the effect of grain size on the mechanical properties of rocks, scholars have conducted meaningful work in uniaxial and conventional triaxial laboratory tests and numerical simulations. Robertson et al. [16] conducted their analysis from rock brittleness, and they found that fine-grained limestone is more brittle than coarse-grained limestone. Scholars have also studied the relationship between the strength and grain size of different types of rocks and concluded that rock strength and grain size show a negative correlation [17,18,19,20,21]. To explain how the grain size affects the uniaxial compressive strength of rocks, Du et al. [22] and Yu et al. [23] carried out uniaxial compression tests on rocks with different grain sizes via indoor tests and numerical simulations, respectively. The results of this study show that the smaller the grain size of the rock, the weaker the stress concentration phenomenon, and its strength is higher.

In addition, the mechanical properties of rocks are also closely related to mineral composition. In the study of the effect of mineral composition on the strength characteristics of rocks, the quartz content has been discussed relatively more. For example, Gunsallus et al. [24] found a positive correlation between the quartz content and uniaxial compressive strength of California granite, and a similar pattern was obtained by Zorlu et al. [25] for sandstone. Some scholars have found that quartz has a limited effect on the strength and deformation of rocks [26,27]. Tandon et al. [28] pointed out that a comprehensive study of the physical, mechanical, mineral composition, and structural properties of rocks in different regions is needed. Some other scholars [29,30] tried to establish a prediction model of uniaxial compressive strength related to the mineral composition of the rock, which provides a reference for analyzing the relationship between rock strength and rock microstructure. According to the analysis of existing studies, the mineral grain size shows a trend of negative correlation with the strength and brittleness; however, the influence of the grain size and mineral composition on the mechanical properties of the rock is still in the stage of qualitative analysis, with no laboratory test considering the influence of the intermediate principal stress.

Since the rock masses in deep engineering are in an environment of triaxial high stress with unequal principal stresses, and the strength, deformation, failure mode, and brittle ductility characteristics of rocks under different true triaxial conditions are different [31,32,33]. A number of true triaxial numerical simulation experiments related to grain size and mineral composition were carried out [34,35,36,37]. For example, Zheng et al. [37] carried out true triaxial numerical simulations of marble with different grain sizes and particle numbers. The experiments showed that the grain size and the number of particles affect the strength of the specimen. However, the numerical simulation method is based on a theoretical framework and assumptions, and the results obtained from numerical simulation have some differences from the real indoor test results.

In order to study the mechanical properties and failure mechanism of marble under the influence of grain size, mineral composition, and medium principal stress, the specimens of marble were taken from China, the Jinping Underground Laboratory Phase II (CJPL-II). The differences in grain size and mineral composition of the marble were analyzed by thin-section analysis and X-Ray Diffraction (XRD) tests. True triaxial laboratory tests were carried out to investigate the stress–strain curves, strength characteristics, deformation characteristics, and brittleness characteristics of marble. The microscopic failure characteristics of the specimens were analyzed through Scanning Electron Microscopy (SEM) and microfracture analysis. In addition, the reasons for the variability in spalling failure in marble are analyzed from a microscopic point of view.

## 2. Materials and Methods

### 2.1. Test Materials

**Figure 1:**
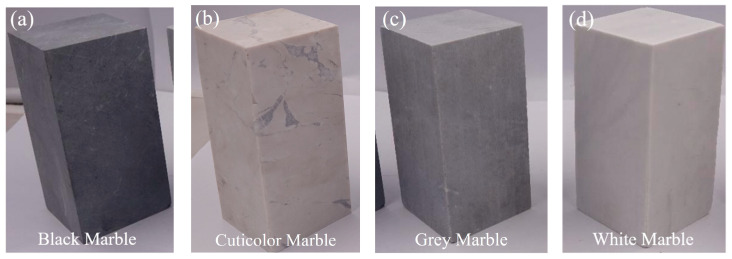
Photographs of Jinping marble in various colors (**a**–**d**).

CJPL-II is located in Jinping Mountain in the lower reaches of Yalong River in Liangshan Prefecture, Sichuan Province, China, with a maximum burial depth of about 2400 m. The rocks are dominated by the mottled marble of Baishan Formation (T_2b_), which is intact and structurally stable. The laboratory adopts a ‘staggered’ arrangement with 4 tunnels and 9 chambers, and the in situ tests of minimum, intermediate, and maximum principal show stress values of 30 MPa, 65 MPa, and 70 MPa, respectively. In order to study the effects of grain size and mineral composition on the mechanical properties and failure mechanism of deep hard rock, several colors of marble, such as black, cuticolor, grey, and white, which are relatively less affected by the geological structure in CJPL-II, were sampled (shown in Figure 1). The test specimen size was a rectangular specimen of 50 × 50 × 100 mm^3^, with dimensions and perpendicularity meeting the requirements of the International Society for Rock Mechanics and Rock Engineering (ISRM) suggested method of true triaxial test [38]. The density, wave velocity, and SEM test methods are based on the ISRM and the standard for test methods of engineering rock mass [39].

### 2.2. Test Methods

In order to obtain the grain size and mineral composition of different marbles, thin-section analysis and XRD tests of the rocks were carried out separately. When performing thin-section analysis, the colored liquid glue was injected into the pores of the original rock under vacuum pressure, and after the liquid gum was cured, it was ground into rock thin sections and observed and analyzed under a microscope. In addition, the rocks were ground into 200-mesh powdered samples, and the rock samples were analyzed using XRD to obtain the mineral composition of the different marbles. The tests were carried out in a Lavender hard rock full stress–strain curve-type true triaxial test system [40]. The test machine can be independently servo-controlled in three directions, with a loading capacity of 3000 kN in the horizontal direction, 6000 kN in the vertical direction, and 100 MPa in the direction of the minimum principal stress. The stiffness of the frame of the test system was evaluated to be 6 MN/mm, which is in compliance with the stiffness requirements of the ISRM test machine [41]. In order to analyze the microscopic failure mechanism of marble in various colors, SEM tests were carried out using an ULTRA PLUS SEM manufactured by Zeiss Microscopy GmbH, München, Germany, with a microscope resolution of 0.8 nm, and in order to obtain the relationship between the microscopic failure characteristics of the specimens and the three-directional stresses, a microfracture analysis of the failure specimens was also carried out on an OLYMPUS BX53.

### 2.3. Test Schemes

**Figure 2:**
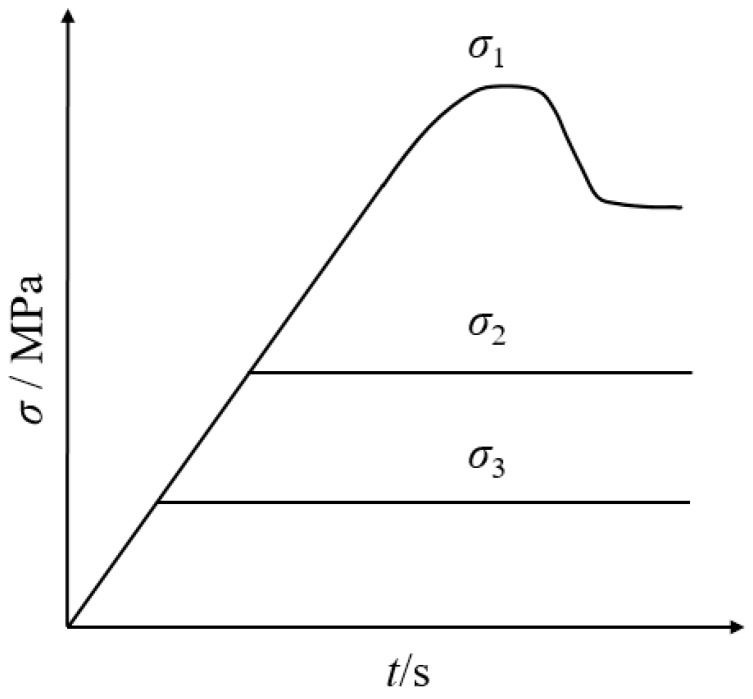
True triaxial loading stress paths.

Under the condition that the minimum principal stress is the in situ stress level (*σ*_3_ = 30 MPa), a loading test of different intermediate principal stresses for different grain sizes and mineral compositions of marble was carried out. The stress paths of the true triaxial loading specimens of marbles in various colors are shown in Figure 2. The minimum principal stress *σ*_3_ was loaded up to 30 MPa; then, the intermediate principal stress *σ*_2_ and the maximum principal stress *σ*_1_ were loaded up to the set value of the intermediate principal stress *σ*_2_, and then *σ*_1_ was loaded individually. When the maximum principal stress *σ*_1_ was loaded up to the vicinity of the peak strength *σ*_p_, the deformation-controlled loading in the direction of the minimum principal stress *σ*_3_ was used instead to obtain the full stress–strain curves.

## 3. Analysis of Results

### 3.1. Microscopic Feature

**Figure 3:**
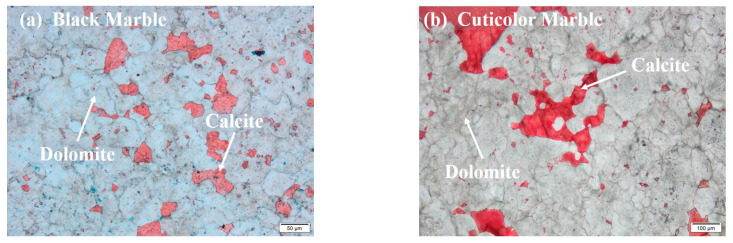

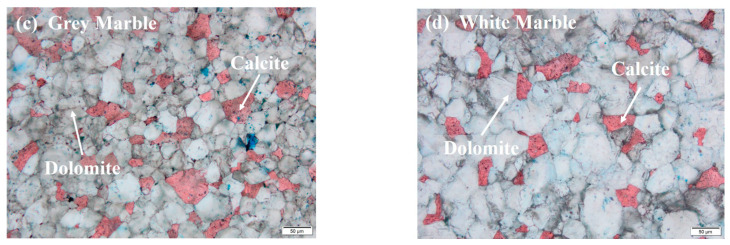
Microscopic characteristics of Jinping marble in various colors.

The microscopic thin-section analysis and XRD results of the specimens before the test are shown in Figure 3 and Table 1, respectively. According to the test results, it can be seen that the various colors of marble are mainly composed of calcite and dolomite. The black marble contains a small amount of quartz, and the cuticolor marble contains a small amount of sodium feldspar. The grain size of black marble, cuticolor marble, grey marble, and white marble is 35–50 μm, 35–125 μm, 35–65 μm, and 35–75 μm, respectively. In addition, all four marbles exhibit a typical granular metamorphic structure, with a close mosaic structure between grains and low porosity. The estimated surface porosity rates of black marble, cuticolor marble, grey marble, and white marble are 1%, 1.5%, 1.5%, and 1%, respectively. Grey marble had locally developed veins, mainly composed of dolomite and calcite, with a coarse grain size, up to 110 μm. The white marble developed several cracks, filled with calcite, with a width of 100–500 μm, and the grain size of the calcite crystals filled in the cracks is mainly 80–150 μm. The specific information of density, wave velocity, and mineral composition of each color marble specimen is shown in Table 1.

### 3.2. Strength Characteristic

**Figure 4:**
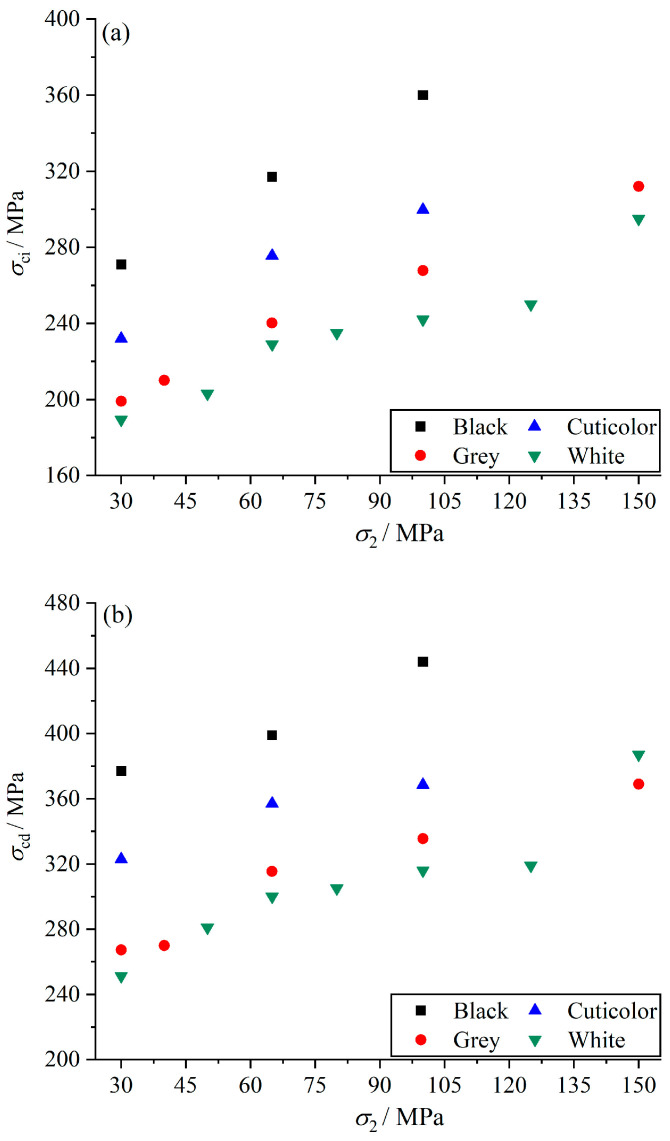

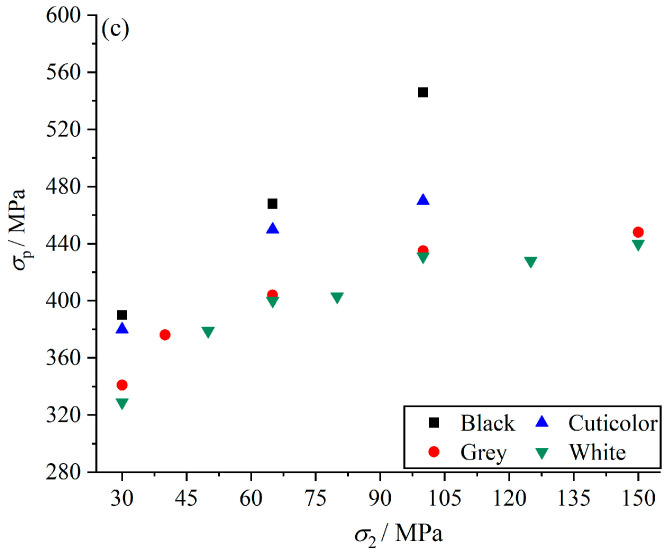
Strength characteristics of marble under different intermediate principal stresses for *σ*_3_ of 30 MPa. (**a**) Crack initiation strength characteristics. (**b**) Damage strength characteristics. (**c**) Peak strength characteristics.

According to the test results, it was found that the grain size, mineral composition, and intermediate principal stress had significant effects on the strength, deformation, and brittle ductility characteristics of the marble. The strength of the specimen is calculated by the Lateral Strain Response (LSR) method [42]. The physico-mechanical properties of marbles in various colors are shown in Table 2. Figure 4a–c show scatter plots of the intermediate principal stress crack initiation strength, intermediate principal stress damage strength, and intermediate principal stress peak strength, respectively. The analysis shows that black marble has the highest strength, cuticolor marble has the second-highest strength, grey marble and white marble are close in strength, and white marble has the lowest strength. In addition, with an increase in the intermediate principal stress, the crack initiation strength, damage strength, and peak strength of each color of Jinping marble increased. At intermediate principal stress of 65 MPa, the crack initiation strength, damage strength, and peak strength of black marble were 15%, 12%, and 4% higher than that of cuticolor marble, 32%, 27%, and 16% higher than that of grey marble, and 38%, 33%, and 17% higher than that of white marble. In addition, at intermediate principal stress of 100 MPa, differences in the strength of different marbles are most significantly affected by grain size and mineral composition. These results are given in Table 2. According to the previous mineral composition and microscopic test results, it can be seen that the particle size of black marble is smaller than that of grey and white marble, and the black marble contains a small amount of quartz, which is harder than the main constituents of marble, dolomite and calcite, so that the strength of black marble is the highest under the action of the same stress conditions. Cuticolor marble varies greatly in grain size but has fewer internal fractures and fillings and contains small amounts of sodium feldspar, which is harder than dolomite and calcite, the main constituents of marble, and is second in strength, only weaker than black marble. Grey and white marbles are similar in grain size and mineral composition, and they also have internal pores and cracks, so their strengths are similar, and both are relatively lower.

The crack initiation stress and damage stress have special physical significance in engineering practice. The crack initiation stress can be used to assess the potential of surrounding rock spalling, while the damage stress is considered to be the upper limit value of the field strength of the surrounding rock, which is used to assess the range of the loosening circle of the surrounding rock. The larger the value of *σ*_ci_/*σ*_p_, the greater the ability of the tunnel surrounding rock to resist the failure of the spalling. The greater the ratio of *σ*_cd_/*σ*_p_, the higher the upper limit of the strength of the surrounding rock at the site. It can be seen in Table 2 that under the same stress conditions, the ratios of *σ*_ci_/*σ*_p_ and *σ*_cd_/*σ*_p_ of black marble are larger, indicating that black marble has the strongest ability to resist spalling failure and the upper limit of surrounding rock strength is higher. The ratio of cuticolor marble is second, and the ratio of grey and white marble is close and the smallest.

### 3.3. Deformation Characteristics

**Figure 5:**
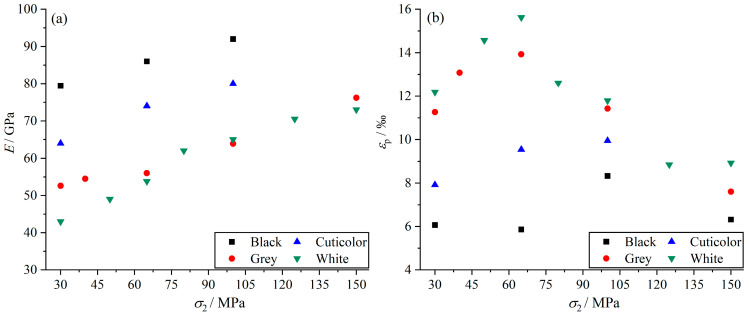
Deformation characteristics of marble under different intermediate principal stress for *σ*_3_ of 30 MPa. (**a**) Elastic modulus characteristics. (**b**) Peak strain characteristics.

The elastic modulus and peak strain of marble with different grain sizes and mineral compositions are shown in Figure 5a,b. The analysis shows that the elastic modulus of marbles in various colors increases with an increase in the intermediate principal stress, and the magnitude of the elastic modulus of marbles in various colors is *E*_B_ > *E*_C_ > *E*_G_ > *E*_W_. Under the same stress conditions, the grey and white marbles with large grain size and containing more pores and cracks have smaller moduli of elasticity and are more prone to deformation than black marble with a smaller grain size and containing high-hardness minerals. Cuticolor marble varies greatly in grain size, but it has fewer internal fractures and fillings and contains small amounts of high-hardness mineral, so the modulus of elasticity or resistance to deformation is intermediate. Under the same stress conditions, the peak strains of marbles in various colors are *ε*_W_ > *ε*_G_ > *ε*_C_ > *ε*_B_. In addition, with an increase in the intermediate principal stress, marbles in various colors show the trend of “increasing first, then decreasing”, the maximum peak strain of grey and white marble occurs when the intermediate principal stress is 65 MPa, and the maximum peak strain of black marble occurs when the intermediate principal stress is about 100 MPa. This indicates that the larger the grain size, the more likely the specimen is to deform, more readily induced by the intermediate principal stress, resulting in the failure of the grey and white marbles occurring faster along the *σ*_2_ direction, and so the peak strain maximum is reached at a lower intermediate principal stress. Black marble has a small grain size and contains a small amount of quartz, making it more difficult to fracture and requiring a larger intermediate principal stress to bring the specimen to its peak strain maximum.

### 3.4. Brittle Ductility Characteristics

**Figure 6:**
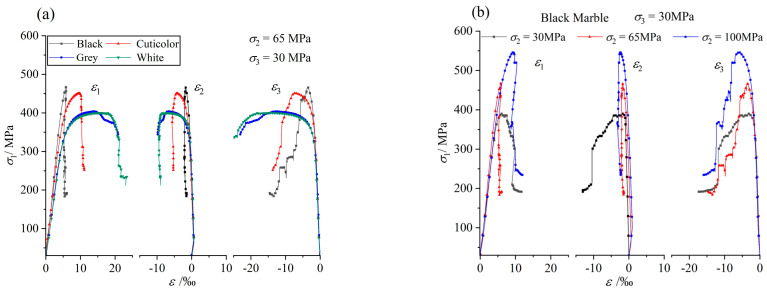

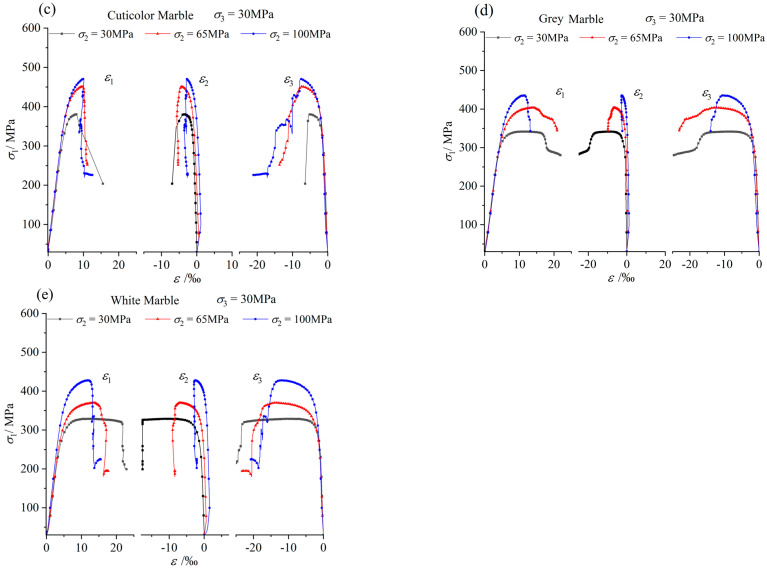
Deformation characteristics of marble under different intermediate principal stresses when *σ*_3_ is 30 MPa. (**a**) Stress–strain curves of marbles in various colors when *σ*_2_ is 65 MPa. (**b**) Stress–strain curves of black marble under different intermediate principal stresses. (**c**) Stress–strain curves of cuticolor marble under different intermediate principal stresses. (**d**) Stress–strain curves of grey marble under different intermediate principal stresses. (**e**) Stress–strain curves of white marble under different intermediate principal stresses.

The full stress–strain curves under different intermediate principal stresses are shown in Figure 6. According to Figure 6a, it can be seen that the brittleness characteristic of black marble is the most obvious. The pre-peak stress–strain curve is close to linear, and the stress drop occurs soon after the peak. The brittleness characteristic of cuticolor marble is the second most obvious, and the pre-peak linear segment is shorter compared with that of the black marble. The post-peak change characteristic is similar to that of black marble, and the stress drop occurs soon. The brittle failure characteristics of grey and white marble are not obvious, large ductile deformation occurs near the peak strength, and the ductile deformation characteristics of white marble are more obvious than that of grey marble. In addition, Figure 6b–e show the full stress–strain curves of black, cuticolor, grey, and white marble under different intermediate principal stresses, respectively. The analysis shows that the brittleness characteristics of marbles in various colors are more significant with an increase in the intermediate principal stress. When the intermediate principal stress increases from 30 MPa to 100 MPa, the brittle failure characteristics of the black and cuticolor marbles become more obvious, while the grey and white marbles change from ductile failure to brittle failure.

**Figure 7:**
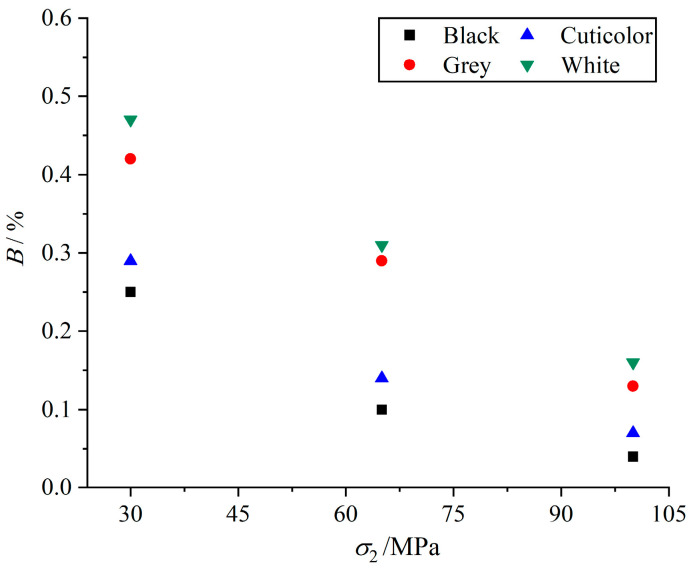
Brittleness index under different intermediate principal stresses for marbles in various colors at *σ*_3_ of 30 MPa.

In order to better analyze the brittle ductility characteristics of marble, we define the brittleness index (*B*) of marbles in various colors and calculation formula as Equation (1), where *ε* is the strain of the specimen when penetration failure occurs; that is, to reach the residual strength of the strain, *ε*_p_ is the peak strain. The calculation of the brittleness index of marbles in various colors with different main stresses of the change rule is shown in Figure 7. The analysis shows that the lower the brittleness index, the more obvious the brittleness characteristics of the specimen, and the brittleness index of marbles in various colors decreases with an increase in the intermediate principal stress. When the intermediate principal stress increases from 30 MPa to 100 MPa, the brittleness indexes of black, cuticolor, grey, and white marbles decrease by 0.21, 0.22, 0.29, and 0.31, respectively, and the brittleness indexes of grey and white marbles decrease more. Since the black and cuticolor marbles already show obvious brittle failures when the intermediate principal stress is 30 MPa, the brittleness indexes have a limited range of variation, while the grey and white marbles show ductile failure, and the brittleness indexes have a greater range of variation.

(1) $$B=(\varepsilon -\varepsilon_{p})/\varepsilon$$

**Figure 8:**
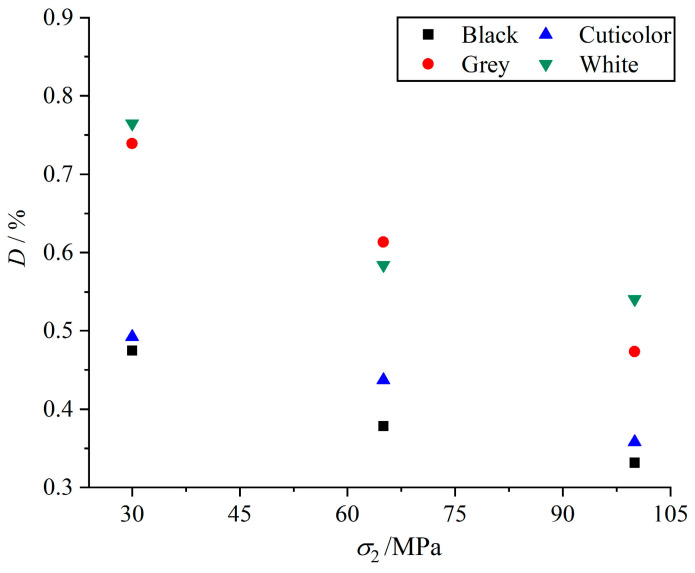
Ductility index under different intermediate principal stresses for marbles in various colors at *σ*_3_ of 30 MPa.

We define the ductility index (*D*) of marbles in various colors and calculation formula as Equation (2), where *ε*_0.9pre-peak_ is the strain that occurs when the specimen reaches 90% of the peak strength before the peak, and *ε*_0.9post-peak_ is the strain that occurs when the specimen reaches 90% of the peak strength after the peak. The change rule of the ductility index of different intermediate principal stresses of marbles in various colors is obtained, as shown in Figure 8. The analysis shows that the higher the ductility index, the more obvious the ductility characteristics of the specimens, and the ductility characteristics of each color of marble decreases with an increase in the intermediate principal stress. When the intermediate principal stress was increased from 30 MPa to 100 MPa, the ductility indexes of black, cuticolor, grey, and white marbles decreased by 0.143, 0.134, 0.265, and 0.224, respectively, and the magnitude of the decrease in the ductility characteristics was higher for grey and white marbles. By calculating the ductility index of the specimens, it is also further illustrated that the brittleness characteristics of grey and white marble have a higher variation amplitude, which can reflect the results of the above analysis for the marble specimens of each color. It shows that the brittleness characteristics of the specimens are negatively correlated with the grain size and positively correlated with the intermediate principal stress. In addition, brittle minerals such as quartz and sodium feldspar can also significantly increase the brittleness characteristics of the specimens.

(2) $$D=(\varepsilon_{0.9\mathrm{post}\text{-}\mathrm{peak}}-\varepsilon_{0.9\mathrm{pre}\text{-}\mathrm{peak}})/\varepsilon_{0.9\mathrm{post}\text{-}\mathrm{peak}}$$

### 3.5. Failure Characteristics

**Figure 9:**
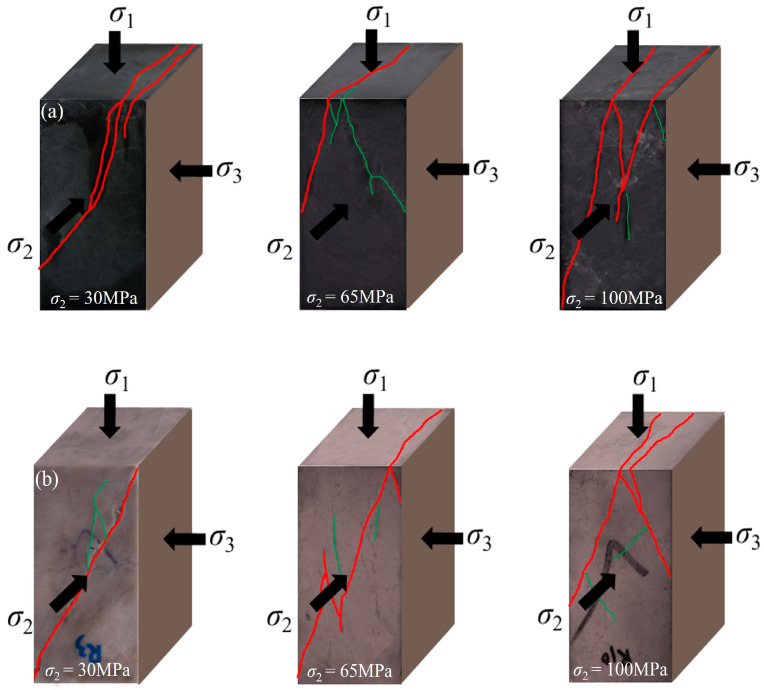

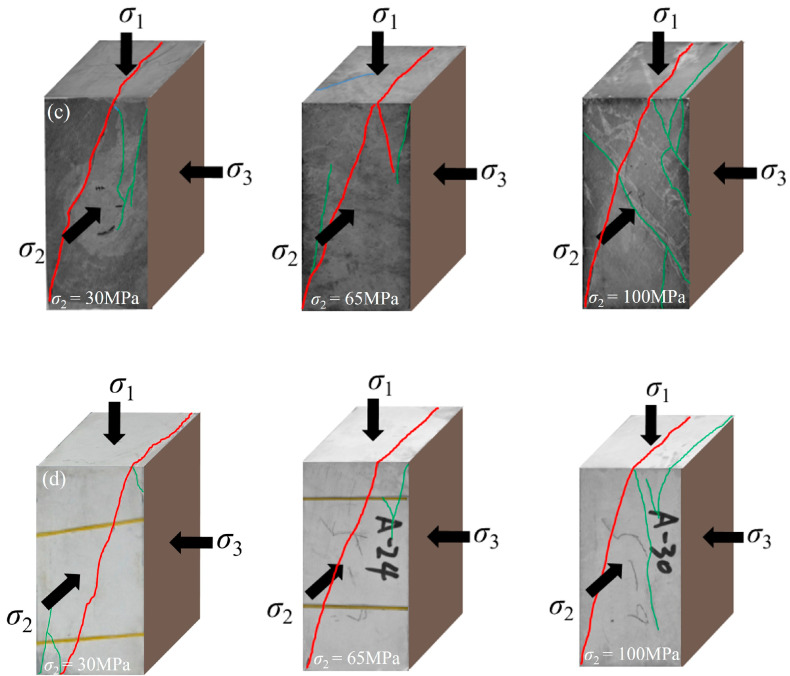
Failure pictures of marbles in various colors with different intermediate principal stresses at *σ*_3_ of 30 MPa. (**a**) Black marble. (**b**) Cuticolor marble. (**c**) Grey marble. (**d**) White marble.

**Figure 10:**
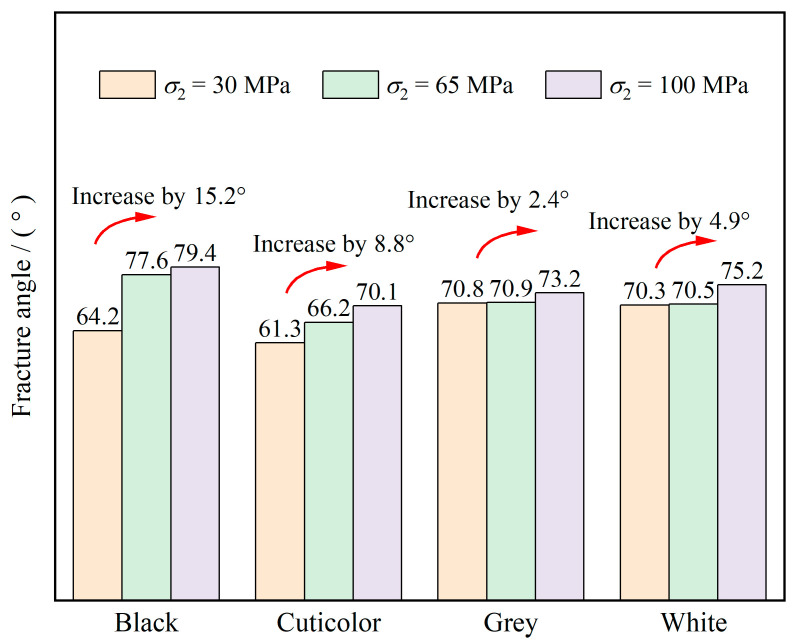
Fracture angle of marbles in various colors under different intermediate principal stresses.

The failure characteristics of marbles in various colors at different intermediate principal stresses when *σ*_3_ is 30 MPa are shown in Figure 9. The angle between the direction of the main cracks of the marble and the horizontal direction is the fracture angle (*β*). The *β* of marbles in various colors at different intermediate principal stress levels is shown in Figure 10. According to the analysis of the test results, the number of vertical microcracks and *β* of marble are significantly affected by the grain size, mineral composition, and intermediate principal stress of marble. In terms of the number of vertical microcracks, black marble with a small grain size is more brittle and has more vertical microcracks, whereas grey and white marble, which are less brittle, have more microcracks along the shear direction. In addition, grey and white marbles appear more densely populated, with microcracks on the macroscopic scale, than black and cuticolor marbles, which also indicates the higher brittleness of black and cuticolor marbles in terms of failure characteristics. In terms of the *β* of marble, it can be found from Figure 10 that the *β* of marbles in various colors has a tendency to increase with an increase in the intermediate principal stress, and the sensitivity of the *β* of marbles in various colors to the intermediate principal stress has a differentiation. The *β* of black marble is larger than that of other colors, and the change in *β* is also higher. When the intermediate principal stress changes from 30 MPa to 100 MPa, the *β* of black marble and grey marble increases by 15.2° and 2.4°, respectively. This indicates that the black marble with a small grain size and containing quartz is more sensitive to the intermediate principal stress, and the grey marble with a large grain size is less sensitive to the intermediate principal stress.

## 4. Failure Mechanisms

### 4.1. SEM Tests

**Figure 11:**
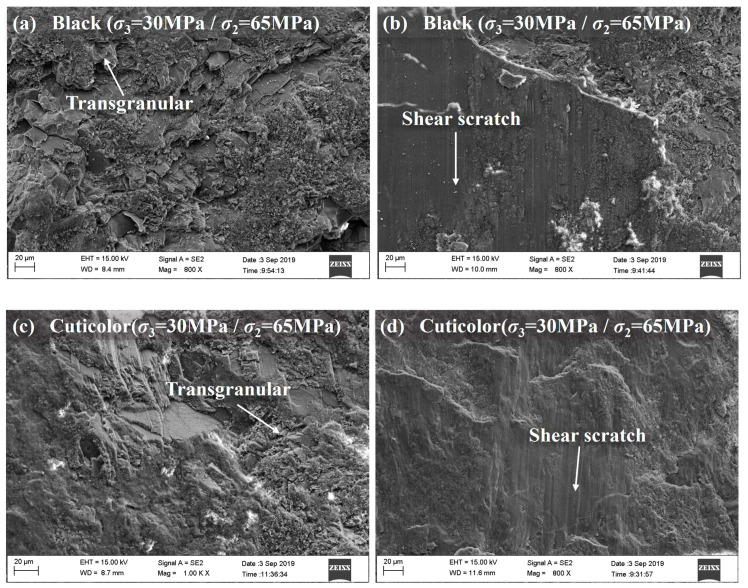

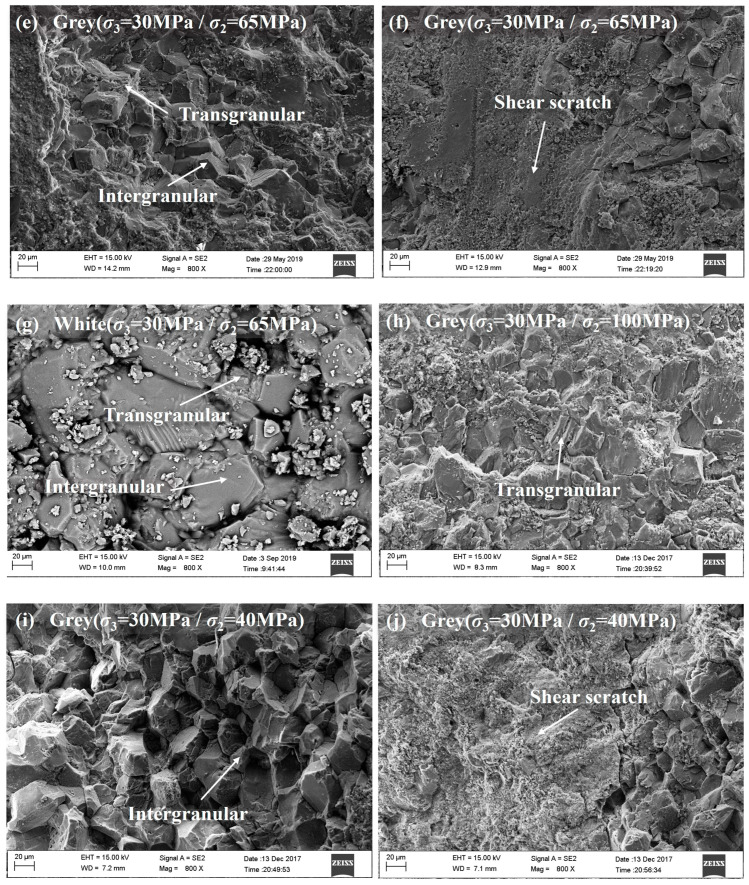
SEM images of marble in various colors (**a**–**j**).

In order to further investigate the failure mechanism of marble with different grain sizes and mineral compositions, SEM tests were carried out on the failure specimens [31,42,43,44], and the test results are shown in Figure 11. Figure 11a–g show the destruction of microscopic features of black, cuticolor, grey, and white marble under the original rock stress condition (*σ*_3_ = 30 MPa/*σ*_2_ = 65 MPa). The overall black marble grain size is smaller, with the most obvious characteristics of transgranular tensile failure. The failure surface has more rock dust, further indicating that the brittle failure of black marble is more intense. As can be seen from Figure 11b, local shear characteristics can be observed in the specimen after failure. As can be seen from Figure 11c,d, the cuticolor marble grain size is larger, with the overall dominance of transgranular tensile failure, and the local shear characteristics of the specimen after failure are also more obvious. As can be seen from Figure 11e,f, grey marble, with a grain size between black and cuticolor, predominantly shows intergranular failure. It exhibits mixed intergranular–transgranular tensile failure characteristics, with shear failure characteristics being the most obvious. In addition, it can be seen from Figure 11g that the failure feature of white marble and grey marble under the same stress condition is similar, and it is a mixed intergranular–transgranular tensile failure.

Figure 11e–j show SEM images of grey marble at intermediate principal stresses of 65 MPa, 40 MPa, and 100 MPa. When the intermediate principal stress is 40 MPa, intergranular failure is predominant, the grain outline is clearly visible, rock powder on the failure surface is not obvious, and local shear failure characteristics exist, as shown in Figure 11i,j. When the intermediate principal stress is 65 MPa, mixed intergranular–transgranular tensile failure is observed to increase, rock powder on the failure surface increases, and shear failure is obvious, as shown in Figure 11e,f. When the intermediate principal stress increases to 100 MPa, the character of transgranular tensile failure increases, the failure surface has more rock powder, and the shear failure is not obvious, as shown in Figure 11h. Overall, with an increase in the intermediate principal stress, grey marble transitions from predominantly intergranular tensile failure, mixed intergranular–transgranular failure, and obvious shear failure to predominantly transgranular tensile failure, with inconspicuous shear failure. The failure of particles becomes more intense, the failure surface of the rock powder gradually increases, and the macroscopic manifestation is the increased presence of microcracks on the specimen.

### 4.2. Microfracture Analysis

**Figure 12:**
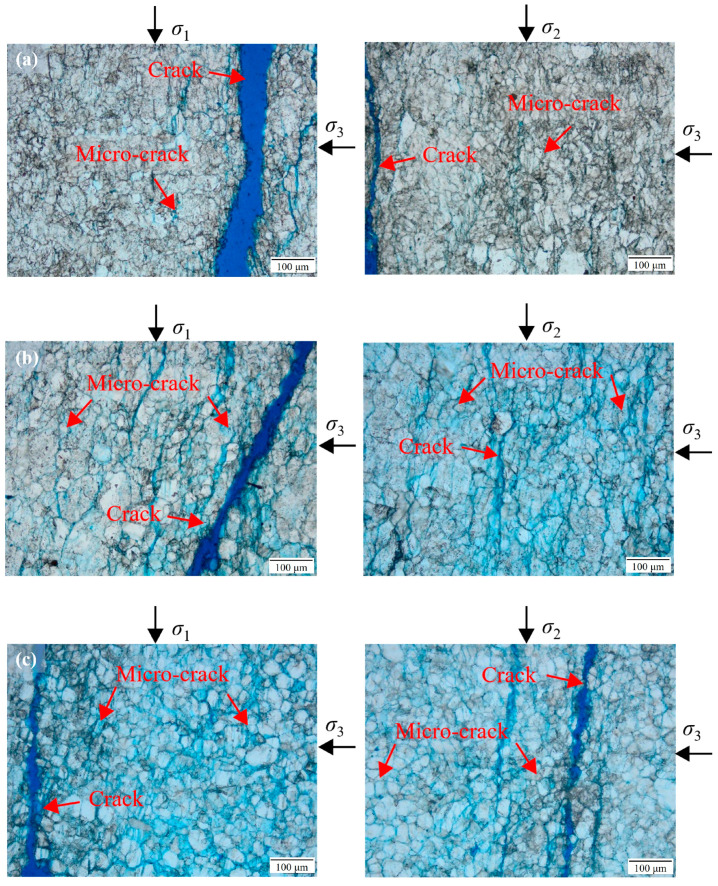

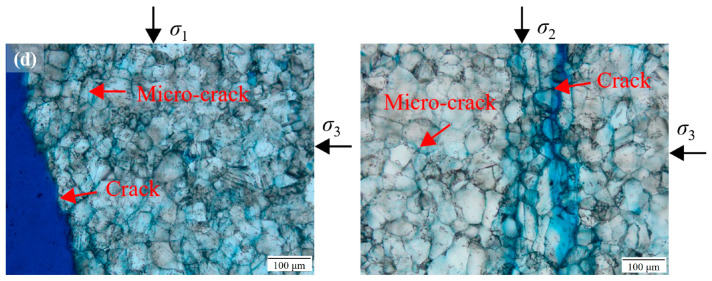
Microfracture analysis results of marbles in various colors at *σ*_3_ = 30 MPa. (**a**) Black marble at *σ*_2_ = 65 MPa; (**b**) cuticolor marble at *σ*_2_ = 65 MPa; (**c**) grey marble at *σ*_2_ = 65 MPa; (**d**) grey marble at *σ*_2_ = 150 MPa.

In order to further analyze the microscopic fracture mechanism of the marble, the failure specimens were infused with resin and sectioned, and microfracture analysis was carried out [3]; the test results are shown in Figure 12. Figure 12a–c show the microfracture analysis results of black, cuticolor, and grey marble under the in situ stress condition (*σ*_3_ = 30 MPa/*σ*_2_ = 65 MPa). It can be seen that the three kinds of marble microcracks, sprouting, expansion, and penetration, are clearly controlled by the stress. The black marble brittleness is stronger, with fewer microcracks present within the specimen. After microcrack formation, rapid expansion leads to the development of obvious macrocracks, while macrocracks surrounding microcrack development are less noticeable. The cuticolor marble has the second-highest brittleness, the obvious microcracks can be seen inside the specimen, and some of the microcracks form and then expand through to obvious macrocracks. The grey marble brittleness is the lowest, and the microcracks inside the specimen are well developed, with the highest number. The extension of local microcracks results in evident macrocracks after their formation, and several macrocracks can be observed. Under the same stress conditions, the number of microcracks shows a negative correlation with the grain size and the content of quartz, sodium feldspar, and other brittle minerals that have higher strength than the marble itself.

In addition, the microfracture analysis results of grey marble under the in situ stress condition (*σ*_3_ = 30 MPa/*σ*_2_ = 150 MPa) are shown in Figure 12d, and compared with the analysis in Figure 12c, it can be seen that the microfracture of grey marble decreases when the intermediate principal stress increases; the brittleness characteristic of the specimen is significantly enhanced, and it is easier for the microfracture to rapidly expand and penetrate after its formation. This is consistent with the results of brittleness characteristics of specimens analyzed in Section 3.3. The characteristics of the specimen showing obvious microcracks when the intermediate principal stress is small are also close to the results after the creep loading of marble, while the characteristics of the specimen microcracks when the intermediate principal stress is large are more similar to the characteristics of the marble when it is transiently loaded [45].

## 5. Discussion and Conclusions

### 5.1. Discussion

**Figure 13:**
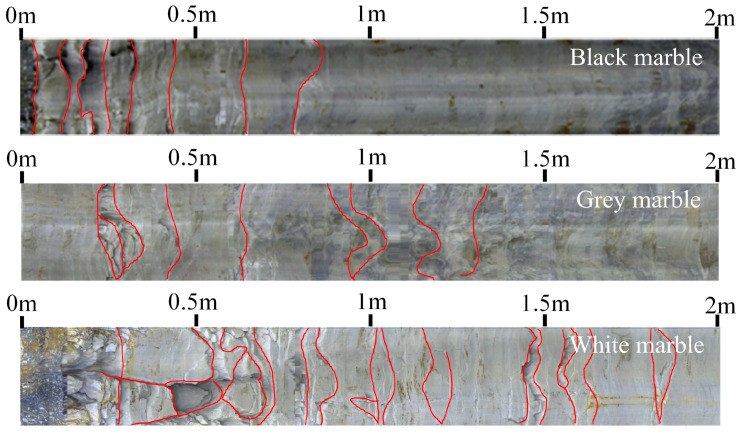
Field borehole images of marbles in various colors sections. The red lines indicate cracks caused by failure.

**Figure 14:**
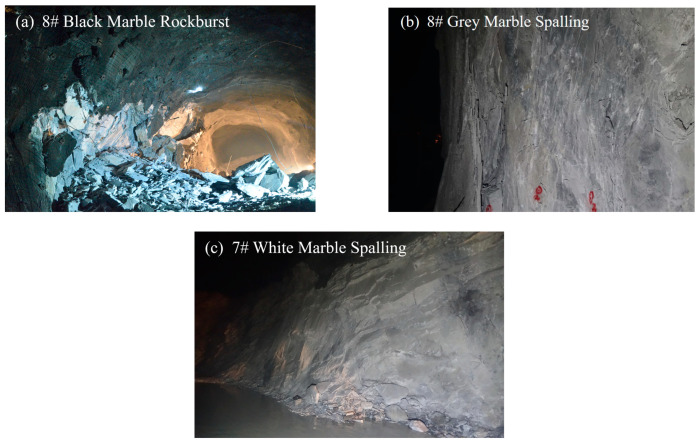
Photographs of failure in the field of marble segments in various colors. (**a**) Black marble rock burst failure; (**b**) grey marble spalling failure; (**c**) white marble spalling failure.

Through field observation of the failure of the Jinping 7–8# underground laboratories, it was found that the depth and degree of spalling of marble in various colors varied under the same geostress conditions. Borehole camera images of the unfolded images of different-colored marbles are shown in Figure 13. As can be seen from the figure, the spalling failure depth of black, grey, and white marble sections gradually increased to 0.8 m, 1.3 m, and 1.9 m, respectively, while the spalling failure degree (that is, the fragmentation degree of the spalling) gradually decreased. Combined with the results of the previous experimental study, it can be seen that the black marble with a small grain size and containing quartz has the highest strength and the shallowest failure depth; the specimen is more brittle, and the spalling failure degree is the largest. The grey and white marble with a large grain size and no quartz has less strength, and the spalling failure in the area where it is located is deeper; the specimens are more ductile, and the failure is smaller. The effect of brittle ductility can also be further verified from the disaster situation in the field. In the more brittle black marble section of Jinping 7–8# underground laboratory, rock burst failures have occurred several times, as shown in Figure 14a. In contrast, in the more ductile grey and white marble section, the spalling failure is more obvious, as shown in Figure 14b,c. The spalling failure of part of the section has also been observed with a clear time-lapse, and the spalling failure is still visible after a few days, still sprouting and developing [13,45].

Through the true triaxial test study of Jinping marble with different grain sizes and mineral compositions, it is known that under the influence of high true triaxial stress in deep underground engineering, the differences in the grain size and mineral composition of the surrounding rock have a more pronounced impact on its deformation and failure. In the future excavation, support, and disaster prevention and control process, it is necessary to comprehensively consider the effects of grain size, mineral composition, and high true triaxial stress.

### 5.2. Conclusions

By carrying out the true triaxial test of Jinping marble with different grain sizes and mineral compositions under the true triaxial stress, the main mechanical properties and failure characteristics of Jinping marble were investigated in relation to grain size, mineral composition, and intermediate principal stress, and the failure mechanism was revealed by combining with XRD, SEM, and microscopic analysis. The main conclusions are as follows:

(1) The crack initiation strength, damage strength, peak strength, and modulus of elasticity of the Jinping marble in various colors increase with an increase in the intermediate principal stress, while the peak strain shows a tendency of increasing and then decreasing with an increase in the intermediate principal stress.
(2) Under the same stress conditions, Jinping marble exhibits increased strength with decreasing grain size, increasing quartz, and sodium feldspar content, following the pattern *σ*_B_ > *σ*_C_ > *σ*_G_ > *σ*_W_, while the peak strain decreases with decreasing grain size and increasing quartz and sodium feldspar content.
(3) The brittleness index *B* and ductility index *D* are defined. The brittleness characteristics and failure angle of Jinping marble are positively correlated with the intermediate principal stress, the content of brittle minerals, such as quartz and sodium feldspar, and negatively correlated with the grain size, while the ductility characteristics are the opposite.
(4) As the intermediate principal stress increases and the grain size decreases, the more pronounced the transgranular tensile failure is and the more violent the failure is in Jinping marble. The number of microcracks increases with increasing grain size and decreases with increasing quartz and sodium feldspar content and intermediate principal stresses.
(5) Under the same geostress conditions, the spalling failure depth in the rock site is positively correlated with the grain size and negatively correlated with the content of brittle minerals, and the spalling failure degree is negatively correlated with the grain size and positively correlated with the content of brittle minerals.

## Figures and Tables

**Table 1:** Basic information of marbles in various colors.

Color	Density (g/cm^3^)	Wave Velocity (m/s)	Mineral Composition (%)				Grain Size
			Dolomite	Calcite	Quartz	Sodium Feldspar	μm
Black	2.82	5300	79	14	7	—	35–50
Cuticolor	2.82	4670	82	17	—	1	35–125
Grey	2.82	4170	80	20	—	—	35–65
White	2.83	4240	85	15	—	—	35–75

**Table 2:** Physical and mechanical properties of marble in various colors.

Marble	No.	*σ*_3_ (MPa)	*σ*_2_ (MPa)	*σ*_ci_ (MPa)	*σ*_cd_ (MPa)	*σ*_p_ (MPa)	*ε*_p_ (‰)	*E* (GPa)	*σ*_ci_/*σ*_p_ (MPa)	*σ*_cd_/*σ*_p_ (MPa)
Black	B1	30	30	271.44	377.03	390.34	6.06	79.46	0.70	0.97
	B2	30	65	317.53	399.15	468.49	5.86	86.21	0.68	0.85
	B3	30	100	360.62	444.70	546.94	8.33	92.14	0.66	0.81
Cuti-color	C1	30	30	232.09	322.93	380.62	7.91	64.75	0.61	0.85
	C2	30	65	275.52	357.95	451.06	9.54	74.26	0.61	0.79
	C3	30	100	299.71	368.65	470.76	9.95	80.19	0.64	0.78
Grey	G1	30	30	199.13	267.29	341.70	11.26	52.59	0.58	0.78
	G2	30	40	210.70	270.09	376.03	13.08	54.57	0.56	0.72
	G3	30	65	240.19	315.39	404.02	13.93	56.03	0.59	0.78
	G4	30	100	267.76	335.66	435.54	11.43	63.88	0.61	0.77
	G5	30	150	312.51	369.47	448.24	7.61	76.24	0.70	0.82
White	W1	30	30	189.32	251.24	328.94	12.18	42.95	0.61	0.76
	W2	30	50	203.18	281.84	378.97	14.57	49.23	0.54	0.74
	W3	30	65	230.46	303.58	400.45	15.62	53.79	0.58	0.76
	W4	30	80	235.01	305.97	403.30	12.61	62.14	0.58	0.76
	W5	30	100	242.18	316.83	428.21	11.79	65.27	0.57	0.74
	W6	30	125	250.74	319.12	428.52	8.85	70.59	0.59	0.74
	W7	30	150	295.34	387.37	440.88	8.92	73.20	0.67	0.88

## Data Availability

Data are contained within the article. The data supporting this study are available on request from the corresponding author.

## References

[1] Haimson B., Chang C. (2000). A new true triaxial cell for testing mechanical properties of rock, and its use to determine rock strength and deformability of Westerly granite. Int. J. Rock Mech. Min. Sci..

[2] Xie H.P., Lu J., Li C.B., Li M.H., Gao M.Z. (2022). Experimental study on the mechanical and failure behaviors of deep rock subjected to true triaxial stress: A review. Int. J. Min. Sci. Technol..

[3] Xu H., Zhang Z., Zhang Y.J., Jiang Q., Qiu S.L., Zhou Y.Y., Feng G.L. (2024). Effects of natural stiff discontinuities on the deformation and failure mechanisms of deep hard rock under true triaxial conditions. Eng. Fail. Anal..

[4] Feng X.T., Xu H., Yang C., Zhang X., Gao Y. (2020). Influence of Loading and Unloading Stress Paths on the Deformation and Failure Features of Jinping Marble under True Triaxial Compression. Rock Mech. Rock Eng..

[5] Xu H., Feng X.T., Yang C., Zhang X., Zhou Y., Wang Z. (2019). Influence of initial stresses and unloading rates on the deformation and failure mechanism of Jinping marble under true triaxial compression. Int. J. Rock Mech. Min. Sci..

[6] Feng G., Ma Q., He Z., Su G., Chen B., Xu D., He J. (2024). Time-delayed failure process of granite and its energy evolution and acoustic emission characteristics. Eng. Fail. Anal..

[7] Si X.F., Huang L.Q., Gong F.Q., Liu X.L., Li X.B. (2020). Experimental investigation on influence of loading rate on rockburst in deep circular tunnel under true-triaxial stress condition. J. Cent. S. Univ..

[8] Feng G.L., Ma J.G., Chen B.R., Xiao Y.X., Jiang Q., Li P.X., Lin M.Q. (2023). Microseismic energy and intensity criterion of rockburst in deep TBM tunnels: A case study of the Neelum-Jhelum hydropower project. J. Cent. South Univ..

[9] Feng X.T. (2017). Rockburst: Mechanisms, Monitoring, Warning, and Mitigation.

[10] Feng G.L., Feng X.T., Chen B.R., Xiao Y.X., Yu Y. (2015). A Microseismic Method for Dynamic Warning of Rockburst Development Processes in Tunnels. Rock Mech. Rock Eng..

[11] Feng G.L., Feng X.T., Chen B.R., Xiao Y.X., Zhao Z.N. (2019). Effects of structural planes on the microseismicity associated with rockburst development processes in deep tunnels of the Jinping-II Hydropower Station, China. Tunn. Undergr. Space Technol..

[12] Read R.S. (2004). 20 years of excavation response studies at AECL’s Underground Research Laboratory. Int. J. Rock Mech. Min. Sci..

[13] Feng X.T., Xu H., Qiu S.L., Li S.J., Yang C.X., Guo H.S., Cheng Y., Gao Y.H. (2018). In situ observation of rock spalling in the deep tunnels of the China Jinping Underground Laboratory (2400 m depth). Rock Mech. Rock Eng..

[14] Mercier-Langevin F., Hadjigeorgiou J. (2011). Towards a better understanding of squeezing potential in hard rock mines. Min. Technol..

[15] Potyondy D.O., Cundall P.A. (2004). A bonded-particle model for rock. Int. J. Rock Mech. Min. Sci..

[16] Robertson E.C. (1955). Experimental study of the strength of rocks. GSA Bull..

[17] Hugman R.H.H., Friedman M. (1979). Effects of texture and composition on mechanical behavior of experimentally deformed carbonate rocks. AAPG Bull..

[18] Wong R.H., Chau K.T., Wang P. (1996). Microcracking and grain size effect in Yuen Long marbles. Int. J. Rock Mech. Min. Sci..

[19] Hatzor Y.H., Palchik V. (1997). The influence of grain size and porosity on crack initiation stress and critical flaw length in dolomites. Int. J. Rock Mech. Min. Sci..

[20] Yılmaz N.G., Goktan R.M., Kibici Y. (2011). Relations between some quantitative petrographic characteristics and mechanical strength properties of granitic building stones. Int. J. Rock Mech. Min. Sci..

[21] Olsson W.A. (1974). Grain size dependence of yield stress in marble. J. Geophys. Res..

[22] Du K., Sun Y., Zhou J., Khandelwal M., Gong F. (2022). Mineral composition and grain size effects on the fracture and acoustic emission (AE) characteristics of rocks under compressive and tensile stress. Rock Mech. Rock Eng..

[23] Yu Q., Zhu W., Ranjith P.G., Shao S. (2018). Numerical simulation and interpretation of the grain size effect on rock strength. Geomech. Geophys. Geo-Energy Geo-Resour..

[24] Gunsallus K.L., Kulhawy F.H. (1984). A comparative evaluation of rock strength measures. Int. J. Rock Mech. Min. Sci..

[25] Zorlu K., Ulusay R.E., Ocakoglu F., Gokceoglu C.A., Sonmez H. (2004). Predicting intact rock properties of selected sandstones using petrographic thin-section data. Int. J. Rock Mech. Min. Sci..

[26] Bell F.G. (1978). The physical and mechanical properties of the fell sandstones, Northumberland, England. Eng. Geol..

[27] Ulusay R., Türeli K., Ider M.H. (1994). Prediction of engineering properties of a selected litharenite sandstone from its petrographic characteristics using correlation and multivariate statistical techniques. Eng. Geol..

[28] Tandon R.S., Gupta V. (2013). The control of mineral constituents and textural characteristics on the petrophysical & mechanical (PM) properties of different rocks of the Himalaya. Eng. Geol..

[29] Zorlu K., Gokceoglu C., Ocakoglu F., Nefeslioglu H.A., Acikalin S.J. (2008). Prediction of uniaxial compressive strength of sandstones using petrography-based models. Eng. Geol..

[30] Yesiloglu-Gultekin N.U., Sezer E.A., Gokceoglu C., Bayhan H. (2013). An application of adaptive neuro fuzzy inference system for estimating the uniaxial compressive strength of certain granitic rocks from their mineral contents. Expert Syst. Appl..

[31] Zhao J., Feng X.T., Zhang X.W., Zhang Y., Zhou Y.Y., Yang C.X. (2018). Brittle-ductile transition and failure mechanism of Jinping marble under true triaxial compression. Eng. Geol..

[32] Feng X.T., Kong R., Zhang X., Yang C. (2019). Experimental study of failure differences in hard rock under true triaxial compression. Rock Mech. Rock Eng..

[33] He M., Ren F., Liu D., Zhang S. (2021). Experimental Study on Strain Burst Characteristics of Sandstone Under True Triaxial Loading and Double Faces Unloading in One Direction. Rock Mech. Rock Eng..

[34] Zhang Q., Zhu H.H., Zhang L.Y. (2015). Studying the effect of non-spherical micro-particles on Hoek-Brown strength parameter m*_i_* using numerical true triaxial compressive tests. Int. J. Numer. Anal. Met..

[35] Xie Y.H., Yang Z.X., Barreto D., Jiang M.D. (2017). The influence of particle geometry and the intermediate stress ratio on the shear behavior of granular materials. Granul. Matter.

[36] Bai Q.S., Young R.P. (2020). Numerical Investigation of the Mechanical and Damage Behaviors of Veined Gneiss During True-Triaxial Stress Path Loading by Simulation of In Situ Conditions. Rock Mech. Rock Eng..

[37] Zheng Z., Tang H., Zhang Q., Pan P., Zhang X., Mei G., Liu Z., Wang W. (2023). True triaxial test and PFC3D-GBM simulation study on mechanical properties and fracture evolution mechanisms of rock under high stresses. Comput. Geotech..

[38] Feng X.T., Haimson B., Li X., Chang C., Ma X., Zhang X., Ingraham M., Suzuki K. (2019). ISRM suggested method: Determining deformation and failure characteristics of rocks subjected to true triaxial compression. Rock Mech. Rock Eng..

[39] (2013). Standard for Test Methods Engineering of Rock Mass.

[40] Feng X.T., Zhang X., Kong R., Wang G. (2016). A Novel Mogi Type True Triaxial Testing Apparatus and Its Use to Obtain Complete Stress–Strain Curves of Hard Rocks. Rock Mech. Rock Eng..

[41] Fairhurst C., Hudson J. (1999). Draft ISRM suggested method for the complete stress-strain curve for intact rock in uniaxial compression. Int. J. Rock Mech. Min. Sci..

[42] Nicksiar M., Martin C.D. (2012). Evaluation of methods for determining crack initiation in compression tests on low-porosity rocks. Rock Mech. Rock Eng..

[43] Qiu S.L., Feng X.T., Xiao J.Q., Zhang C.Q. (2014). An Experimental Study on the Pre-Peak Unloading Damage Evolution of Marble. Rock Mech. Rock Eng..

[44] He Z., Li G., Tian S., Wang H., Shen Z., Li J. (2016). SEM analysis on rock failure mechanism by supercritical CO_2_ jet impingement. J. Pet. Sci. Eng..

[45] Feng X.T., Yang C.X. (2023). Hard Rock Mechanics in Deep Engineering.

